# Assessing the Risk of Probiotic Dietary Supplements in the Context of Antibiotic Resistance

**DOI:** 10.3389/fmicb.2017.00908

**Published:** 2017-05-19

**Authors:** Min Zheng, Ruijia Zhang, Xuechen Tian, Xuan Zhou, Xutong Pan, Aloysius Wong

**Affiliations:** College of Natural, Applied and Health Sciences, Wenzhou-Kean UniversityWenzhou, China

**Keywords:** antibiotic resistance, probiotics, *Lactobacillus*, dietary supplements, probiotic supplements

## Abstract

Probiotic bacteria are known to harbor intrinsic and mobile genetic elements that confer resistance to a wide variety of antibiotics. Their high amounts in dietary supplements can establish a reservoir of antibiotic resistant genes in the human gut. These resistant genes can be transferred to pathogens that share the same intestinal habitat thus resulting in serious clinical ramifications. While antibiotic resistance of probiotic bacteria from food, human and animal sources have been well-documented, the resistant profiles of probiotics from dietary supplements have only been recently studied. These products are consumed with increasing regularity due to their health claims that include the improvement of intestinal health and immune response as well as prevention of acute and antibiotic-associated diarrhea and cancer; but, a comprehensive risk assessment on the spread of resistant genes to human health is lacking. Here, we highlight recent reports of antibiotic resistance of probiotic bacteria isolated from dietary supplements, and propose complementary strategies that can shed light on the risks of consuming such products in the context of a global widespread of antibiotic resistance. In concomitant with a broader screening of antibiotic resistance in probiotic supplements is the use of computational simulations, live imaging and functional genomics to harvest knowledge on the evolutionary behavior, adaptations and dynamics of probiotics studied in conditions that best represent the human gut including in the presence of antibiotics. The underlying goal is to enable the health benefits of probiotics to be exploited in a responsible manner and with minimal risk to human health.

## Introduction

Probiotics are “live microorganisms which when administered in adequate amounts confer a health benefit on the host” (Kaur et al., 2002). The concept of ‘probiotics’ and the idea of using these friendly bacteria to enhance health and delay the process of aging was first proposed by the Russian scientist, Elie Metchnikoff more than a century ago (Mackowiak, 2013). Research has since showed that probiotics can confer a wide range of health benefits especially those directly related to the human gut (Nagpal et al., 2012; Kechagia et al., 2013). For instance, probiotics can regulate gut microbiota (Thomas et al., 2014), improve immune system (Isolauri et al., 2001) and the bioavailability of nutrients (Scholz-Ahrens et al., 2007), reduce symptoms of lactose intolerance (Savaiano et al., 2013) as well as prevent and/or treat gastrointestinal infections (Parvez et al., 2006; Ringel et al., 2012). Other notable health claims include the prevention of mammary cancer (Lakritz et al., 2014), the reduction of viral-associated pulmonary damage (Zelaya et al., 2014) and the decrease in cholesterol level that reduces the risk of cardiovascular diseases (Ejtahed et al., 2011).

Of interest to this article is the probiotic bacteria that are present in commercial dietary supplements. This category of functional foods consists of millions to billions of commercially manufactured probiotic bacteria and often a heterogenous population of probiotics packed in one tablet or capsule thus representing the largest amount of probiotics consumed by people across all food categories. In light of the escalating global impact of antibiotic resistance, dietary supplements containing such high amounts probiotics represent an excellent condition for the spread of resistant determinants especially when sharing residence with intestinal microflora and opportunistic pathogens in the human gut (Broaders et al., 2013).

**FIGURE 1:**
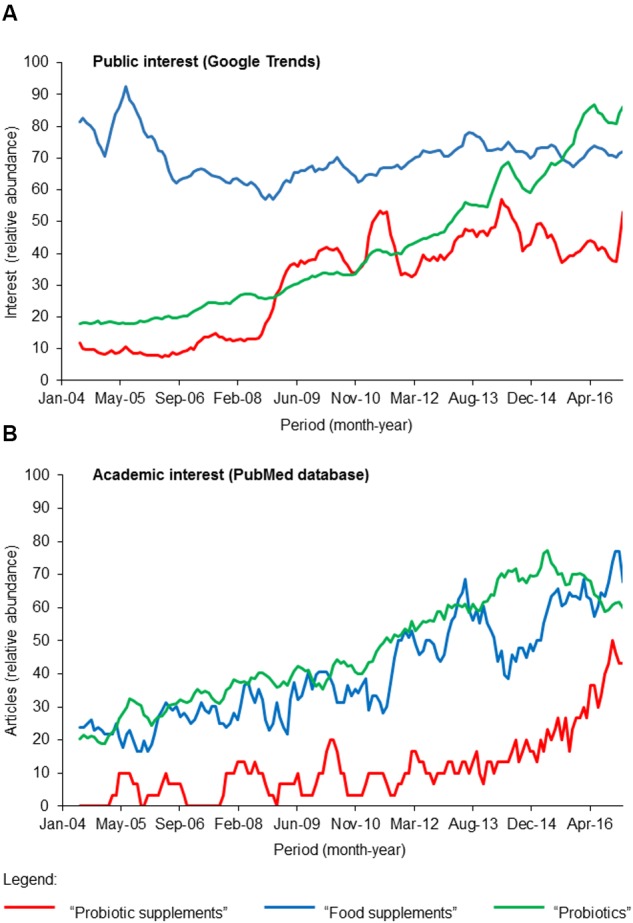
**A survey of (A)** public and **(B)** academic interests of “probiotic supplements” (red), “food supplements” (blue), and “probiotics” (green) within the past 15 years (2004-February 2017). The keywords “probiotic supplements,” “food supplements,” and “probiotics” were used in the survey of public interest using Google Trends (https://trends.google.com/trends/) and to retrieve scholarly articles indexed in the PubMed database. The number of articles and Google Trend hits are normalized against the highest values of the respective search terms within the period between 2004 and February 2017.

The 2015 global probiotic market size by revenue exceeded 35 billion USD^1^. According to Grand View Research, Inc. (2017), the “probiotics dietary supplement” segment represents the second largest segment of probiotics market by revenue after the “probiotic food and beverage” segment and is projected to grow at a CAGR of 7.5% over the forecast period of 2014–2024. In concomitant with the growth of probiotics global market is the public interest and this is reflected in the steady increased in Google searches (search term: “probiotics”) in the last 15 years (**Figure 1A**) while interest in “food supplements” is relatively constant in the same period. Importantly, interest in “probiotic supplements” jumped 20% within a short period (2008–2009) and stayed relatively stable thereafter. This marked increase in public interest corresponds well with the recent market growth of probiotic supplements thus implying that this category of functional food is gaining global popularity. As such, there is reciprocal interest in academics as the same “probiotic supplements” search term yielded an exponential increase in scholarly outputs from 2012 according to records in the PubMed database (**Figure 1B**). Taken together, this analysis suggests that while probiotic supplements are gaining popularity, research on this relatively new segment of functional food is lagging.

Here, we highlight recent reports of antibiotic resistance detected from probiotic bacteria isolated from this increasingly popular but understudied source of probiotics, and propose complementary strategies that can shed light on the risk of consuming such products in the context of the global widespread of antibiotic resistance.

## A Brief Account of Probiotics and Antibiotic Resistance

Since probiotics are known to confer a plethora of health benefits some of which have been supported by scientific evidence, it is therefore not surprising that they are now included in foods such as dairy products like yogurt, cheese and milk as well as dietary supplements (Homayouni et al., 2012; Nagpal et al., 2012). Further, the addition of probiotics in animal feed formulations have enabled their uptake into the human gut through the food chain (Sharma et al., 2014). Despite the many health benefits of probiotics, there are increasing concerns regarding the long-term safety of consuming probiotics or probiotics-fortified foods that remain unaddressed therefore posing a “double-edged” dilemma on their usage (Imperial and Ibana, 2016). Among the concerns that threaten the safety of probiotics are the occurrence of diseases such as bacteremia or endocarditis, the release of toxic or causing negative metabolic effects on the gastrointestinal tract and the potential transfer of antibiotic resistant determinants in the human gut (Snydman, 2008; Broaders et al., 2013); the latter being the primary focus of this article.

In modern medicine, antibiotics are the “panacea” for all infections and will likely be the mainstay of our defense against microbial infections for the foreseeable future. However, the increasing reliant on antibiotics coupled with malpractice, misuse and abuse of antibiotics, have laid heavy selective pressure on pathogenic bacteria which have inevitably but adversely accelerated the otherwise gradual evolutionary process of bacteria adaptation to antibiotics due in large parts to the increased frequency in the transfer of resistant genes carried on mobile elements. In line with the increasing inclusion of probiotics in foods as well as their escalating global demand, it is imperative that the benefits of probiotics are harnessed without contributing to the spread of antibiotic resistance. Probiotics and specifically the lactic acid bacteria are generally regarded as safe as reflected by their GRAS status. While probiotics that harbor resistant genes are themselves not harmful (Kaur et al., 2002), the potential transfer of resistance genes from probiotics to other gut microflora and eventually to opportunistic pathogens that share the same intestinal habitat, may, however, pose serious clinical ramifications (Broaders et al., 2013; Gueimonde et al., 2013; Imperial and Ibana, 2016).

Since the observation of the transfer of resistant gene carried on plasmid from Shigella to *E. coli* in 1959 (Harada et al., 1961), many resistant determinants in clinical bacteria have been attributed to some sort of transfer event rather than by mutation. This is due to the substantial sequence differences between resistant genes as observed in for, e.g., the β-lactamases and aminoglycoside inactivating enzymes (Bush et al., 1995), thus making evolution by mutation alone seemingly impossible within a relatively short time frame (Davies, 1997). Molecular studies have revealed that gene exchange among bacteria, and between bacteria and animal or human hosts occurring in soil and the human gut, is a more plausible model to explain the origins of these resistant determinants in pathogenic bacteria many of which resemble those found in naturally occurring antibiotic producing microorganisms. This shows that human interactions with bacteria observed traditionally in agricultural and veterinary activities and more recently in food preparations and commercial probiotic formulations, can facilitate the uptake of resistant determinants from a diverse pool of resistant genes already present in the environment and the gut (Davies, 1997; Broaders et al., 2013). Bacteria can transfer genes horizontally through conjugation, natural transformation or transduction and these mechanisms have been reviewed elsewhere (Thomas and Nielsen, 2005; Huddleston, 2014). Of these mechanisms, conjugative transfer of genes involving transposons and integrons are most interesting because they allow genes to shuttle promiscuously within the genome and also between the genome and the circular plasmids thereby overcoming limitations of species boundaries (Hall, 2012).

Antibiotic resistant determinants have been detected in bacteria isolated from various sources including hospitals, human samples, animal feeds, water treatment plants and foods. Importantly, probiotic isolates from dairy products, starter cultures and probiotic-containing foods as well as from human origins and animal feeds have also been reported to harbor antibiotic resistant determinants (Charteris et al., 1998a,b, 2001; Teuber et al., 1999; Sidhu et al., 2001; Mathur and Singh, 2005; Kastner et al., 2006; Ocana et al., 2006; Aquilanti et al., 2007; Liu et al., 2009; Zonenschain et al., 2009; Botina et al., 2011; Drago et al., 2011; Nawaz et al., 2011; Gueimonde et al., 2013; Sharma et al., 2014). However, the antibiotic resistant profiles of commercial probiotic bacteria from the increasingly popular food category, dietary supplements, have remain scarce (Wong et al., 2015). Due to the widespread usage, genes that confer resistance to the respective classes of antibiotics in *Lactobacillus* have been characterized (Kastner et al., 2006; Aquilanti et al., 2007; Nawaz et al., 2011; Gueimonde et al., 2013). For instance, a recent study has identified the *van(X)*, *van(E)*, *gyr(A)*, and *tet(M)* genes that code for resistance to the respective vancomycin, ciprofloxacin and tetracycline antibiotics (Guo et al., 2017). Another study on *Lactobacillus* showed that resistance to oxytetracycline, clindamycin, and/or erythromycin are contributed by the respective *erm(B)* and/or *tet(W)*, *tet(M)* genes (Klare et al., 2007) while *aac(6′)-aph(2′)-Ia* is found in *Lactobacilli* that are resistant to gentamicin. Those containing *tet(L)*, *tet(M)* and *tet(S)* are resistant to tetracycline while the *aadA* gene codes for resistance to streptomycin and spectinomycin (Bujnakova et al., 2014).

Elucidating the antibiotic resistant profiles of probiotic bacteria from different sources including dietary supplements, will enable the understanding of how these bacteria device strategies to protect themselves from the destructive actions of antibiotics. For instance, bacteria can alter the target sites of antibiotics or lower the concentration of free antibiotics in the cell by the action of efflux pumps which together with the outer membrane barrier, efficiently excludes antibiotics. Some bacteria achieve resistance by producing enzymes (e.g., β-lactamase) to degrade antibiotics while others (e.g., penicillin-binding proteins) structurally alter the antibiotics target sites thus rendering them ineffective. A detailed account of mechanisms of antibiotic resistance have been reviewed (Hawkey, 1998; Cox and Wright, 2013; Sharma et al., 2016).

## Probiotic Supplements: Antibiotic Resistance Among Other Concerns

Despite the health benefits, probiotic bacteria isolated from several commercially available dietary supplements have recently been reported to harbor resistance toward multiple antibiotics (Wong et al., 2015). The authors reported probiotics that showed resistance toward different classes of antibiotics including glycopeptides (vancomycin), aminoglycosides (streptomycin and gentamicin), mono-bactams (aztreonam) and fluoroquinolones (ciprofloxacin) all of which, are broad spectrum antibiotics known to be effective against Gram positive and Gram negative bacteria. This preliminary study presents a cause for concern because antibiotic resistant determinants have been previously demonstrated to be able to transfer from one *Lactobacillus* to another and importantly, also from *Lactobacilli* to other species including pathogens such as *Staphylococcus* (Tannock et al., 1994) and vice versa (Mater et al., 2008). For instance, the transfer of a vancomycin resistance gene from *Enterococci* to a *Lactobacillus acidophilus* commercial strain was observed *in vitro* and importantly also *in vivo* in the gut of mice at high frequencies (Mater et al., 2008). Therefore, recent reports that encouraged the development of protective commensal bacteria as next-generation probiotics (Buffie and Pamer, 2013; Pamer, 2016) should be approached with caution. While probiotics can restore the immediate intestinal microbiota post-antibiotic treatment, re-establish colonization resistance and eliminate potential pathogens, a build-up of antibiotic resistant genes in the gut may present longer term consequences that outweigh these immediate benefits. Follow-up studies that include behavioral and evolutionary characterizations of probiotic commensal bacteria under antibiotic pressure should be employed since this practice may lead to increased *trans*-conjugation (Gueimonde et al., 2013; Wong et al., 2015; Imperial and Ibana, 2016). Since the transfer of resistant determinants occur at high frequencies in mouse models, it is highly likely that such events may also occur in the human gut given that they act as reservoir for antibiotic resistant genes (Broaders et al., 2013). Given the risks of resistant gene transfer and accumulation in the gut, other non-antibiotic approaches to prevent or eliminate pathogens (Nigam et al., 2014) could be adopted in place of probiotics. These alternatives include the use of phages (Sulakvelidze et al., 2001; Letchumanan et al., 2016), bactericidal peptides (Cleveland et al., 2001), killing factors released by microbes (Liu et al., 2010), non-antibiotic drugs (Amaral and Kristiansen, 2000) and quorum quenching (Carnes et al., 2010).

Since some health claims are not substantiated by scientific evidence, these supplements are not regulated as drugs by the U.S. Food and Drug Administration (FDA) therefore enabling these probiotic-rich products to be sold over-the-counter without prescription. Indeed, some exaggerated health claims have been previously challenged and subsequently dropped from the product labels^2^. This has inevitably tainted the field with controversies as specific health claims will naturally be approached with distrust and discord (Pamer, 2016). While direct scientific evidence for the use of probiotics in reducing infections and enhancing certain body functions are lagging (Przyrembel, 2001), the commercial interests and exploitations have rapidly expanded in recent years (**Figure 1**). Due to the promise of rich commercial gains and to a certain extent the lack of clear legislations, some manufacturers have perhaps unethically made exaggerated claims in the product labels to compete for consumers. Common issues with product labels of probiotic supplements include the overestimation of live bacteria, the misidentification of probiotic strains and their associated health claims (Berner and O’Donnell, 1998; Hamilton-Miller et al., 1999; Farnworth, 2008; Wong et al., 2015). In light of the limited but confounding scientific data and in line with increasing commercial and public interests, future research efforts should be centered on understanding the long term impact of consuming probiotic dietary supplements and the danger posed by antibiotic resistance. In the following section, we propose how the preliminary screenings of antibiotic resistance in these supplements can be followed up with research works that will improve our current understanding (or the lack thereof) on both the near- and far-reaching effects of long-term consumption of high amounts of probiotic bacteria.

## Assessing the Risk of Antibiotic Resistance in Probiotics of Dietary Supplements

**FIGURE 2:**
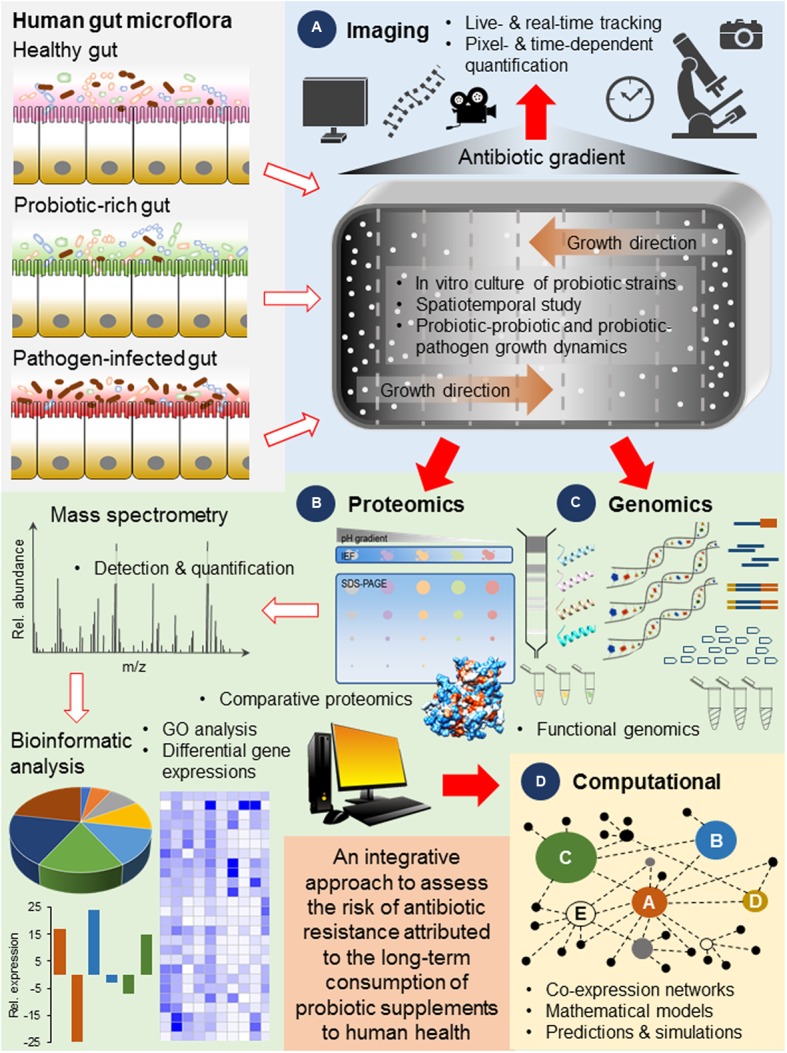
**A proposed scheme of workflow illustrating the use of (A)** live imaging technologies to visualize and track the growth and evolution of probiotic bacteria in real-time (Baym et al., 2016), **(B,C)** advanced molecular tools for the broad coverage and high resolution system level detection and gene expression analysis (Marondedze et al., 2014) and **(D)** powerful computational approaches for the formation of predictive models simulating the behavior of probiotic bacteria based on laboratory data (Waclaw, 2016), for the assessment of the risk of antibiotic resistance contributed by commercial probiotic strains and their long-term consumption to human health. This integrative approach should be applied in studies that consider the different compositions of the gut microflora such as the presence of a consortia of probiotic strains and pathogens as well as a gradient of antibiotic pressures, and their growth and colonization dynamics in response to various spatial and temporal factors.

Currently, a pilot study on antibiotic resistance of probiotics were conducted on several popular brands of dietary supplements manufactured in the United States, Austria and Malaysia (Wong et al., 2015). The authors used the de Man, Rogosa and Sharpe (MRS) media to selectively culture *Lactobacillus* probiotic strains thus resistance profiles of other probiotic strains were omitted from the screenings. These screenings should not only be expanded to other brands of commercial probiotic supplements but also include other probiotic strains such as *Bifidobacteria*, *Enterococcus*, and *Streptococcus*. More importantly, these probiotic bacteria should be characterized to determine their resistant determinants using a system level approach such as deep sequencing and comparative proteomics and studied in *in vitro* conditions that mimic their natural environment in the gut in the presence or absence of antibiotics (**Figure 2**). Comparative proteomics (Fouhy et al., 2015; Casado Munoz Mdel et al., 2016; Perez-Llarena and Bou, 2016) and functional genomics (Hughes, 2003; Cottell et al., 2014; Suzuki et al., 2014) are increasingly used to elucidate novel genes related to antibiotic resistance and infer biological functions to previously uncharacterized proteins in bacteria including probiotics (**Figures 2B,C**). A global understanding of the regulatory networks underpinning genetic events such as the physiological networks that dictate bacteria evolution and the metabolic rewiring that lead to resistance is necessary given that the tight regulation of resistant determinants and the ability to trigger transient phenotypic antibiotic resistance are key strategies for bacteria to reduce susceptibility to antibiotics under different circumstances (Martinez et al., 2009).

Importantly and unlike conventional evolutionary studies employing well-mixed systems such as the stirred flasks or bioreactors, a method that allows for direct and real-time visualization of mutation and selection in a migrating bacterial front can be adopted. For instance, a recent study has demonstrated microbial evolution dynamics over a large antibiotic landscape on an experimental device known as the microbial evolution and growth arena (MEGA) plate (Baym et al., 2016). This custom-built dish has an area (120 cm × 60 cm) large enough to accommodate successive regions of increasing antibiotic where bacteria first inoculated at one region of no antibiotic, are allowed to spread by chemotaxis across spatially varying antibiotic pressure and colonize new regions when they deplete the nutrients locally. Additionally, current advances in live imaging tools (Son et al., 2015) and analytical programs (Schindelin et al., 2012) have enabled pixel- and time-dependent quantification of bacterial growth to be studied in higher resolution (**Figure 2A**). Interestingly, this innovative device when used in tandem with modern genomics and proteomics tools, has revealed new insights concerning bacteria mutational diversification, adaptation rates and assigned new biological functions to genes involved in bacteria adaptation and colonization efficiency. The genetic profiles of more adaptable colonizers were also determined and such molecular data, can shed light on how probiotics behave and evolve when under antibiotic pressure, when interacting with other gut microflora and when in direct interaction with pathogens since highly resistant mutants may become overspecialized and are trapped behind the propagating front (Baym et al., 2016).

Further, the molecular strategies determined from the system level analysis of probiotic bacteria colonies at the propagating front can be used to generate hypothesis and form mathematical models to predict the evolutionary behavior of commercially manufactured probiotic strains in the spatiotemporal environment of the gut and simulate their interactions with the gut microflora, with other probiotic strains and with pathogenic strains in the presence of antibiotics (**Figure 2D**). For instance, computational models revealed that bacteria may switch to an alternative phenotype [e.g., consuming a different food source (Solopova et al., 2014)] that is less sensitive to a new and unfavorable environmental condition, can afford bacteria time to develop compensatory mutations that offset the effect of the deleterious mutation and regain fitness (Greulich et al., 2012; Waclaw, 2016). The use of mathematical models to simulate how phenotypic switching can provide a larger pool of bacteria to evolve genetic resistance to antibiotics has been previously reviewed (Levin and Rozen, 2006). The concomitant integration of laboratory data with computational models to predict adaptive and evolutionary strategies of commercially manufactured probiotic bacteria can therefore lead to the manufacturing of safer dietary supplements and can assist health practitioners in determining suitable prescriptions when evaluating the course of antibiotics to be undertaken by patients. Such comprehensive data and rigorous assessments would enable a clear set of regulations to be mapped out to guide manufacturers and medical practitioners as well as to better inform consumers on both the benefits and risks associated with the consumption of probiotic dietary supplements. The underlying goal is to enable the many promising health benefits of probiotic bacteria to be exploited in a responsible manner and with minimal risk to long-term human health.

## Author Contributions

AW conceived this project, wrote the manuscript and made the figures; MZ and RZ wrote the manuscript, performed data analysis and made the figures; XT, XZ, and XP contributed to the writing of the manuscript; All authors read and approved the final version of the manuscript.

## Conflict of Interest Statement

The authors declare that the research was conducted in the absence of any commercial or financial relationships that could be construed as a potential conflict of interest.

## Footnotes

1 http://www.grandviewresearch.com/industry-analysis/probiotics-market

2 https://www.ftc.gov/news-events/press-releases/2010/12/dannon-agrees-drop-exaggerated-health-claims-activia-yogurt
